# Occupational Physical Activity and Body Mass Index: Results from the Hispanic Community Health Study / Study of Latinos

**DOI:** 10.1371/journal.pone.0152339

**Published:** 2016-03-31

**Authors:** Richard H. Singer, Mark Stoutenberg, Marc D. Gellman, Edward Archer, Sonia M. Davis, Nathan Gotman, David X. Marquez, Christina Buelna, Yu Deng, H. Dean Hosgood, Ruth E. Zambrana

**Affiliations:** 1 Department of Public Health Sciences, University of Miami Miller School of Medicine, Miami, Florida, United States of America; 2 College of Dental Medicine, Nova Southeastern University, Ft. Lauderdale, Florida, United States of America; 3 Department of Psychology, University of Miami, Miami, Florida United Sates of America; 4 Office of Energetics, Nutrition Obesity Research Center, University of Alabama Birmingham, Birmingham, Alabama, United States of America; 5 Department of Biostatistics, Gillings School of Global Public Health, University of North Carolina - Chapel Hill, Chapel Hill, North Carolina, United States of America; 6 Department of Kinesiology and Nutrition, University of Illinois at Chicago, Chicago, Illinois, United States of America; 7 Institute for Behavioral and Community Health, Graduate School of Public Health, San Diego State University, San Diego, California, United States of America; 8 Department of Epidemiology and Population Health, Albert Einstein College of Medicine, Bronx, New York, United States of America; 9 Department of Women's Studies, Director, Consortium on Race, Gender and Ethnicity, University of Maryland, College Park, Maryland, United States of America; University of Tennessee Health Science Center, UNITED STATES

## Abstract

**Purpose:**

To examine the associations between overweight/obesity and occupation among Hispanics/Latinos, the largest minority population in the U.S.

**Methods:**

This study included 7,409 employed individuals in the Hispanic Community Health Study/Study of Latinos (HCHS/SOL), a prospective study of Hispanic/Latino individuals aged 18–74 in four communities in the U.S. We independently examined the relationships between BMI, Occupational Activity (OA), and Total Hours Worked, quantified via self-reported hours worked per week and occupation-assigned Metabolic Equivalents (METs).

**Results:**

More than three quarters of the participants were either overweight (39.3%) or obese (37.8%). Individuals with a primary occupation and those employed in a secondary occupation worked an average of 36.8 and 14.6 hrs/wk, respectively. The overall adjusted odds for being obese compared to normal weight were 3.2% (AOR = 1.03, 95% CI 1.01, 1.05) and 14.4% (AOR = 1.14 95% Cl 1.07, 1.23) greater for each 10 MET•hrs/wk unit of increased OA, and each 10-hrs/wk unit of Total Hours Worked, respectively.

**Conclusion:**

This study presents the first findings on the association between OA with overweight/obesity among Hispanic/Latino individuals in the U.S. Increasing OA and Total Hours Worked per week were independently associated with increasing odds of overweight/obesity suggesting that the workplace is only one part of the overall energy expenditure dynamic. Our findings point to the need to emphasize engaging employed individuals in greater levels of PA outside of the work environment to impact overweight/obesity.

## Introduction

The negative health consequences associated with the “obesity epidemic” have been well documented and present a significant impact upon public health [1,2]. Nearly one-third of the U.S. adult population is obese and the prevalence among many minorities is even higher [3]. For men and women together, the prevalence of obesity is greater for Hispanic/Latino (28.7%) and non-Hispanic black (35.7%) adults, than for non-Hispanic white adults (23.7%) [3]. The association of obesity with hypertension, hyperlipidemia, and diabetes signifies that obese individuals are particularly at risk of death from CVD and its sequelae [4,5]. Yet, despite the public health impact associated with increasing incidence of obesity, identification of the factors leading to this phenomenon are still undetermined and have yet to be fully examined in Hispanic/Latino populations.

Obesity is the result of a chronic positive energy balance achieved by consuming more energy than is expended (weight change = energy consumed−energy expended). The primary modifiable variable of the expenditure component is physical activity (PA) that is categorized into four domains: occupational, transportation, household, and leisure-time activities. Considering that leisure-time physical activity (LTPA) has not changed significantly over the period that the prevalence of obesity has increased [6,7], most research has focused on the energy intake (consumption) component of the model in an attempt to explain the rise in U.S. adult obesity [8–10]. Recent questioning of the validity of population-level energy intake data [11,12] has led to increasing attempts to quantify other domains of energy expenditure [13–15].

Based upon observations that time spent in LTPA represent a relatively small proportion of our waking hours and that a large segment of waking hours are spent at work, Church et al. [15] developed a conceptual framework that argued that occupational PA has a greater potential impact than LTPA on daily total energy expenditure. He reported that over the past 50 years in the U.S., the progressive decrease in the percent of individuals employed in moderate intensity occupations resulted in a reduction of >100 calories in daily energy expenditure. A positive energy balance as little as 50 kcal/day can lead to an average gradual weight gain of 1–2 pounds per year [16–18]. Accordingly, the reduction in occupational PA advanced by Church et al. [15] accounted for a significant proportion of the increase in mean body weight among U.S. men and women.

Existing literature presents conflicting findings regarding the association between occupational energy expenditure and Body Mass Index (BMI) [19–27]. In some instances [19,26,27] results are consistent with those of Church et al., [15] namely, that lower BMIs were associated with occupations involving moderate to high levels of PA. Other studies [20–24] have reported an inverse relationship between BMI and occupational level, where higher BMIs were associated with higher energy expenditure occupations. Still others report no association [25,28]. Gender differences appear to contribute further to the controversy [21–24] among employed individuals.

Data from the U.S. Department of Labor, Bureau of Labor Statistics [29] point to disparities in occupational patterns between Hispanic and non-Hispanic segments of the U.S. population. A lower percent of Hispanics were employed in professional or management related occupations and a larger percent were employed in trade or production occupations than the total and non-Hispanic white populations. Such disparities may point to unique associations between occupational PA and the risk of being overweight and obese in Hispanic/Latino populations. While previous studies have examined the relationship between BMI and occupation in Thai [19], Dutch [21], Spanish [22], Australian [23], Canadian [24], Polish [25], and general U.S. populations [26], to our knowledge a focused examination of the relationship between BMI and employment status or occupational PA has not been conducted in the U.S. Hispanic/Latino population. Identification of occupations that convey risk of overweight or obesity in the Hispanic/Latino population may provide opportunities to identify ethnically appropriate strategies in the workplace that may be used for health promotion and prevention of obesity. Given the increasing prevalence of obesity and its associated morbidity, mortality, and economic impact, our principal aim is to obtain an understanding of the manner in which occupational PA contributes to, or protects against, the obesity epidemic in a Hispanic/Latino population.

## Methods

### Participants

The current study used data acquired in the Hispanic Community Health Study/Study of Latinos (HCHS/SOL). The HCHS/SOL is a prospective study that enrolled 16,415 self-identified Hispanic/Latino individuals aged 18–74 in four U.S. communities (Bronx, Chicago, Miami, San Diego) from diverse cultural origins. Recruitment, which began in 2008, was implemented through a two-stage area household probability design [30].

The HCHS/SOL is conducted under the oversight of the Institutional Review Boards (IRB) at each of the following participating institutions constituting the regional field centers: Albert Einstein College of Medicine/Montefiore Medical Center; Feinberg School of Medicine, Northwestern University in collaboration with the Midwest Latino Health Research Training & Policy Center at the University of Illinois at Chicago; Miller School of Medicine of the University of Miami; San Diego State University Graduate School of Public Health; and the HCHS/SOL Coordinating Center at the University of North Carolina. The participants provided written informed consent, in either Spanish or English, prior to data collection. Specific details regarding sample design, cohort selection and implementation of the HCHS/SOL have been previously described [30,31]. The study examination included multiple interviewer-administered questionnaires, available in both Spanish and English.

### Body Mass Index

The dependent variable in this study, BMI, was calculated from direct measurements of participant height and weight and was categorized according to National Institute of Health (NHLBI/NIH) guidelines [32] as follows: BMI <18.5 = underweight; 18.5 ≤ BMI ≤ 24.9 = normal weight; 25 ≤ BMI ≤ 29.9 = overweight; and BMI ≥ 30 = obese. This categorization permitted the evaluation of differences among underweight, normal, overweight, and obese individuals, where normal weight served as the reference category.

### Occupational Physical Activity

Measures of occupational PA included, Employment Status, Weekly Occupational Activity (OA) and Self-Reported Occupational Activity (SROA). Employment Status was self-reported as currently employed (full-time or part-time) or unemployed. All other occupational measures were limited to the subset of employed individuals. Employment was self-described as full-time (≥ 40 hours/week) or part-time (< 40 hrs/wk). Occupations were categorized into one of 13 HCHS/SOL Occupational Categories included in our data set (S1 Table). The primary occupation was defined as the one in which individuals spent the most time. For people with more than one job, a secondary occupational category was also recorded. Equating the 13 occupational categories with activity levels was based upon the assignment of metabolic equivalent (MET) values attributed to the 2002 Census Occupational Classification System (OCS) [33] by Tudor-Locke et al., [34] and followed by Church et al. [15]. Each key word and descriptor of the 13 self-reported HCHS/SOL occupational categories was cross-matched to the 509 detailed occupations in the OCS. For each HCHS/SOL occupational category (Table 1), the assigned MET value was calculated as the average of the cross-matched MET equivalents from the OCS database, to each of the descriptors from the HCHS/SOL occupational categories. Weekly OA (MET•hrs/wk) was calculated as the product of: (1) the assigned MET value of the stated occupational category and (2) the hours worked per week in that occupation. For individuals with two jobs, the total Weekly OA was the sum of the Weekly OA for both occupations. For example, Weekly OA for an individual employed 8 hours per day, 6 days per week as a Senior professional (assigned value of 1.77 METs) was calculated as (1.77 MET) x (8hr/day) x (6 day/week) = 70.8 MET•hrs/wk. A continuous variable describing *SROA* (hrs/wk) was constructed from the sum of self-reported time spent in moderate occupational PA, such as carrying light loads or brisk walking, and vigorous occupational PA, such as carrying heavy loads, digging or construction work, weighted by 2 [35,36]. Total Hours Worked (hrs/wk) was a continuous variable defined for each individual as all of the hours worked per week across all jobs.

**Table 1:** Occupational MET* Categorical Assignment Scheme (N = 7,336^§^).

Range	Occupation	MET	Row % (SE)	Row% (SE)
1.00–1.99	Office staff	1.60	4.99 (0.45)	
	Senior professional / technical worker	1.77	1.90 (0.24)	
	Administrator / executive / manager	1.93	4.04 (0.51)	
	Driver	1.93	2.71 (0.28)	13.64 (0.86)
2.00–2.99	Athlete, actor, musician	2.44	0.58 (0.20)	
	Junior professional / technical worker	2.50	2.96 (0.29)	
	Army officer, police officer	2.50	0.24 (0.08)	
	Ordinary soldier, policeman, fireman	2.58	0.18 (0.08)	
	Service worker	2.64	17.69 (0.80)	
	Skilled worker	2.81	23.49 (0.82)	45.14 (0.89)
3.00–3.99	Other	3.11	12.87 (0.66)	12.87 (0.66)
≥ 4.00	Non-skilled worker	4.39	28.29 (0.95)	
	Farmer, fisherman, hunter, lumberjack	5.00	0.05 (0.03)	28.34 (0.95)

* MET—Metabolic Equivalent of Task (kcal/kg*hr)

^**§**^ 7,336 individuals had non-missing values for METs and all covariates.

### Covariates

Leisure-time Physical Activity (LTPA) was constructed categorically as “none”, “moderate”, “vigorous”, or “moderate and vigorous”, based upon participant responses to the questions, “Do you do any vigorous-intensity (or moderate-intensity) sports, fitness or recreational (leisure) activities that cause large (or small) increases in breathing or heart rate for at least 10 minutes continuously?” for vigorous and moderate LTPA, respectively. Transportation Physical Activity (TPA) was a categorical variable coded as “no” or “yes”, where “yes” referred to walking or bicycling continuously for at least 10 minutes to get from place to place. Energy Consumption was a derived variable based on two 24-Hour Dietary Recalls that were administered in-person at the field center, with a second dietary recall being administered at least five days after the initial examination (and ideally within 45 days). Total energy intake was predicted using a one-part nonlinear mixed model specified by the NCI method using single component statistical macros developed at the NCI [37,38]. Socioeconomic Status (SES) was based upon Annual Household Income in the HCHS/SOL Economic Questionnaire. Hispanic Background was self-reported as either, Dominican or Dominican descent, Central American or Central American descent, Cuban or Cuban descent, Mexican or Mexican descent, Puerto Rican Puerto Rican descent, South American or South American descent, or more than one heritage/other. Acculturation was defined using the 6-question Short Acculturation Scale for Hispanics (SASH) language acculturation subscale and the 4-question SASH social acculturation subscale, each consisting of 5-point Likert-type responses with 1 = Only Spanish / All Hispanic/Latino and 5 = Only English / All non-Hispanic/non-Latino. The degree of acculturation of participants is indicated by the averages of the 6 language subscale questions and 4 social subscale questions respectively. *Marital Status* was categorized as single, married or living with a partner, and separated, divorced or widowed. Cigarette Use was described by all participants, as never, former, or current smokers.

### Statistical Analysis

Statistical analyses were conducted using SAS 9.4 software (SAS Institute, Cary, NC). The methodology employed for all analyses accounted for the complex sampling design, and applied the overall normalized sampling weights that were calibrated to the U.S. 2010 Census within the HCHS/SOL target areas, such that estimates reported generalize to the Hispanic/Latino population age 18–74 living in these geographic areas [30,31]. Univariate analyses were performed to provide summary statistics of the characteristics of the analytic sample (Table 2). Bivariate associations for selected independent variables within the 4-level BMI classification, referenced to individuals with normal BMIs, were performed using unadjusted Rao-Scott Chi-Square tests for categorical variables and t-tests for comparing means of continuous variables (S2 Table). Multivariable polytomous survey logistic regression analysis of BMI categories were conducted to compute adjusted odds ratios (AOR) for BMI categories compared to normal BMI. Each model included a primary independent variable of interest, namely, Employment Status, SROA, Weekly OA, and Total Hours Worked, as well as the control covariates (age, income, Hispanic/Latino background, acculturation, cigarette use, dietary energy consumption, as well as TPA and LTPA). Moreover, stratification of the multivariable analyses was performed by domain analysis for employment (i.e., employed vs. unemployed) and gender in order to permit valid estimates of sample variance. P-values were not adjusted for multiple comparisons.

**Table 2:** Demographic characteristics of employed individuals participating in the Hispanic Community Health Study/Study of Latinos (HCHS/SOL), United States 2008–2011 (n = 7,409). Unadjusted column percent (95% CL), Mean (SE).

	Overall		Overall
Univariate Characteristic	(N = 7,409)*	Univariate Characteristic	(N = 7,409)*
	Col. Percent (95% CL)		Col. Percent (95% CL)
	Mean (SE)		Mean (SE)
**GENDER**		**PRIMARY OCCUPATION**^§^	
Male	56.24 (54.56, 57.92)	Office staff	4.94 (4.06, 5.82)
Female	43.76 (42.08, 45.44)	Senior professional/technical worker	1.88 (1.42, 2.35)
		Administrator/executive/manager	4.01 (3.01, 5.00)
**AGE**	38.95 (0.25)	Driver	2.68 (2.13, 3.23)
18–29 years	26.95 (25.13, 28.77)	Athlete, actor, musician	0.58 (0.19, 0.96)
30–39 years	25.78 (24.02, 27.55)	Junior professional/Technical worker	2.93 (2.37, 3.48)
40–49 years	25.30 (23.93, 26.66)	Army officer, police officer	0.24 (0.09, 0.39)
50–59 years	15.67 (14.66, 16.68)	Ordinary soldier, policeman	0.18 (0.01, 0.34)
60–69 years	5.68 (4.96, 6.40)	Service worker	17.53 (15.98, 19.09)
70–74 years	0.62 (0.38, 0.86)	Skilled worker	23.28 (21.68, 24.87)
		Other^c^	12.76 (11.47, 14.05)
**EDUCATION**^a^		Non-skilled worker	28.03 (26.20, 29.86)
< HS	14.96 (13.75, 16.17)	Farmer, fisherman, hunter	0.05 (0.01, 0.10)
HS (or Preparatory School)	39.12 (37.07, 41.18)	Don’t know/refused	0.91 (0.57, 1.24)
Trade or Vocational School	11.36 (10.11, 12.61)		
University or College	32.92 (30.83, 35.01)	**PRIMARY OCCUPATION** (hrs/wk)	36.77 (0.23)
Other	1.63 (1.20, 2.06)		
		**SECONDARY OCCUPATION**^§^	
**HOUSEHOLD INCOME**		Office staff	1.50 (0.44, 2.57)
< $10,000	7.32 (6.39, 8.24)	Senior professional/technical worker	1.73 (0.60, 2.85)
$10,001–20,000	27.77 (25.85, 29.70)	Administrator/executive/manager	3.67 (1.59, 5.76)
$20,001–40,000	37.99 (36.19, 39.79)	Driver	1.11 (0.19, 2.03)
$40,001–75,000	18.85 (17.18, 20.52)	Athlete, actor, musician	2.06 (1.03, 3.09)
> $75,000	8.07 (6.48, 9.67)	Junior professional/Technical worker	2.53 (1.15, 3.92)
		Army officer, police officer	0.24 (0.00, 0.52)
**MARITAL STATUS**^b^		Ordinary soldier, policeman	0.47 (0.00, 1.21)
Single	33.06 (31.22, 34.91)	Service worker	17.51 (13.69, 21.34)
Married or living with a partner	53.33 (51.27, 55.39)	Skilled worker	19.11 (14.44, 23.77)
Separated, divorced, or widow(er)	13.61 (12.50, 14.71)	Other^c^	17.35 (12.54, 22.15)
		Non-skilled worker	24.26 (18.44, 30.08)
**BMI**	29.10 (0.11)	Farmer, fisherman, hunter	0.16 (0.00, 0.40)
Underweight (<18.5)	0.75 (0.41, 1.10)	Don’t know/refused	8.30 (2.84, 13.76)
Normal (18.5–24.9)	22.16 (20.78, 23.54)		
Overweight (25.0–29.9)	39.30 (37.65, 40.95)	**SECONDARY OCCUPATION** (hrs/wk)	14.61 (0.57)
Obese (≥ 30)	37.79 (35.98, 39.60)		
		**TOTAL HOURS WORKED** (hrs/wk)	38.33 (0.24)
**ACCULTURATION**			
Language Subscale	2.15 (0.03)	**EMPLOYMENT**	
Language Subscale	2.27 (0.01)	Part-time (< 40 hrs/wk)	39.28 (37.51, 41.06)
		Full-Time (≥ 40 hrs/wk)	60.72 (58.94, 62.49)
**BACKGROUND**		**PHYSICAL ACTIVITY**	
Dominican	9.42 (7.85, 11.00)	Leisure Time (≥ 10 continuous min)	
Central American	8.26 (6.89, 9.63)	None	54.61 (52.49, 56.72)
Cuban	15.57 (12.60, 18.55)	Moderate	16.16 (15.03, 17.28)
Mexican	43.52 (40.02, 47.02)	Vigorous	13.37 (11.97, 14.78)
Puerto Rican	12.70 (11.16, 14.24)	Moderate & Vigorous	15.86 (14.24, 17.49)
South American	6.03 (5.10, 6.97)	Occupational Energy Expenditure (kcal/wk)	9,849.24 (96.01)
More than one/Other heritage	4.49 (3.58, 5.40)	Occupational Activity (MET*hrs/wk)	117.33 (1.02)
		Self-Reported Occupational Physical Activity (hrs/wk)	38.27 (0.87)
**CIGARETTE USE**		Transportation (walk or bicycle ≥ 10min)	
Never	64.12 (62.37, 65.88)	No	55.08 (52.90, 57.25)
Former	17.75 (16.55, 18.94)	Yes	44.92 (42.75, 47.10)
Current	18.13 (16.68, 19.59)	**DIETARY COMPOSITION**	
		Total Energy Consumption (kcal/day)	2,074.73 (9.93)

Note: Total number of participants in the HCHS/SOL was 16,415 individuals, of these 8,259 (50.31%) were not currently employed; the characteristics of the employed participants with non-missing data for Age, BMI, Income, Background, Acculturation, Cigarette Use, Energy Consumed, Transportation Physical Activity, and Leisure Time Physical Activity (N = 7,409, 45.14%) are presented above.

^**§**^ Occupations in order of increasing MET equivalents; Secondary Occupation refers to employment in addition to the primary occupation (i.e., a second job).

^a.^ Responses missing for 34 individuals

^b.^ Responses missing for 7 individuals

^c.^ MET equivalent for occupation “Other” was the population based weighted mean of all other occupations.

## Results

Approximately half of the 16,415 participants in the HCHS/SOL sample were employed (N = 8,156). Covariate data was missing for 747 (9.2%) of the employed individuals that resulted in their exclusion from the analysis and produced a final analytic sample of 7,409. Population estimates of the demographics, employment characteristics, PA domains, and total caloric consumption are presented in Table 2. The mean age was 38.9 years, 56.2% were male, and 53.3% were married or living with a partner. Individuals of Mexican descent comprised the largest estimated component of the population (44%), followed by individuals of Cuban (16%) and Puerto Rican (13%) backgrounds. Seventy-three percent of the household incomes were $40,000 or less. The population was predominantly non-smokers (64%), with 18% former smokers. The mean BMI was 29.1 kg/m^2^, with more than three-quarters of the participants either overweight (39.3%) or obese (37.8%).

The majority of the employed individuals were employed full-time (60.7%), with the remainder employed part-time (39.3%). Individuals with a single occupation worked an average of 36.8 hrs/wk and those with a second occupation, an estimated 10.6% of the population, worked a mean of 14.6 additional hrs/wk. Overall, the estimated mean total hours worked was 38.8 hrs/wk. Less than half (44.9%) walked or bicycled for transportation, and greater than half (54.6%) responded that they did not participate in LTPA.

The population estimated associations between BMI categories and employment status inferred from the entire HCHS/SOL sample with non-missing covariates (N = 14,114) are displayed in Table 3. After adjusting for the covariates, the odds of being underweight compared to normal weight were 47.9% lower (AOR = 0.521, 95% CI 0.289, 0.940) in employed compared to unemployed individuals. No other main effects were observed between BMI categories and employment status in this model. Moreover, no significant associations between BMI category and employment status were identified after stratification by gender.

**Table 3:** Categorical polytomous BMI outcomes regressed upon Employment Status (Employed vs. Unemployed) among individuals participating in the HCHS/SOL study, stratified by gender (N = 14,114^a^).

	Overall				Female				Male			
	(N = 14,114)				(N = 8,342)				(N = 5,772)			
	Prev^b^	AOR	95% CI	p-value	Prev^b^	AOR	95% CI	p-value	Prev^b^	AOR	95% CI	p-value
**Underweight** (< 18.5)	0.75	0.521	(0.289, 0.940)	0.030	0.73	0.486	(0.225, 1.047)	0.065	0.78	0.665	(0.333, 1.330)	0.249
**Normal** (18.5–24.9)	18.97	1	1	…	18.83	1	1	…	19.18	1	1	…
**Overweight** (25.0–29.9)	37.86	1.071	(0.924, 1.241)	0.363	34.54	0.985	(0.819, 1.186)	0.877	42.65	1.052	(0.852, 1.299)	0.639
**Obese** (≥ 30)	42.42	0.956	(0.824, 1.110)	0.556	45.90	0.935	(0.774, 1.130)	0.486	37.39	0.866	(0.695, 1.079)	0.200

Variables forced into the model as covariates: Age, Income, Background, Acculturation, Cigarette Use, Energy Consumed, Transportation Physical Activity, and Leisure Time Physical Activity.

^a.^ 2,301 individuals were missing values for some or all covariates.

^b.^ Adjusted prevalence.

The model presented in Table 4 presents the relationship between BMI categories and SROA (hrs/wk). After accounting for the covariates, there were no significant overall or gender-specific associations with BMI.

**Table 4:** Categorical polytomous BMI outcomes regressed upon Self-Reported Occupational Activity^§^ among employed individuals participating in the HCHS / SOL study, stratified by gender (N = 3,951^a^).

	Overall				Female				Male			
	(N = 3,951)				(N = 1,797)				(N = 2,154)			
	Prev^b^	AOR	95% CI	p-value	Prev^b^	AOR	95% CI	p-value	Prev^b^	AOR	95% CI	p-value
**Underweight** (< 18.5)	0.81	0.991	(0.977, 1.006)	0.258	1.00	1.003	(0.980, 1.027)	0.782	0.70	0.988	(0.970, 1.007)	0.206
**Normal** (18.5–24.9)	22.47	1	1	…	24.33	1	1	…	21.41	1	1	…
**Overweight** (25.0–29.9)	39.53	1.001	(0.998, 1.004)	0.520	34.05	1.001	(0.994, 1.007)	0.825	42.65	1.001	(0.997, 1.004)	0.783
**Obese** (≥ 30)	37.19	1.003	(1.000, 1.007)	0.061	40.61	1.005	(0.999, 1.011)	0.120	35.23	1.002	(0.998, 1.006)	0.307

^§^ Self-Reported Occupational Activity (SROA) reported in units of hours x week^-1^

AOR—Adjusted Odds Ratio.

Variables forced into the model as covariates: Age, Income, Background, Acculturation, Cigarette Use, Energy Consumed, Transportation Physical Activity, and Leisure Time Physical Activity.

^a.^ 3,951 individuals had non-missing values for SROA and all covariates.

^b.^ Adjusted prevalence.

Table 5 shows the association between BMI categories and Weekly OA, described in units of 10 MET•hrs/wk. After adjusting for the covariates, the overall adjusted odds for being overweight were 3.8% greater than the odds of being normal weight for each 10 MET•hrs/wk unit of increased Weekly OA (AOR = 1.04, 95% CI 1.02, 1.06). Similarly, there was a 3.2% increased odds of being obese compared to normal weight for each 10 unit increase in MET•hrs/wk (AOR = 1.03, 95% CI 1.01, 1.05). Males exhibited trends similar to the overall population with a 4.5% and 4.0% increased risk of being overweight (AOR = 1.05, 95% CI 1.02, 1.07) or obese (AOR = 1.04, 95% CI 1.01, 1.07) vs. normal weight, respectively, for each increasing 10 MET•hours/wk of Weekly OA. There were no significant associations for females when examining the relationship between Weekly OA and BMI. Moreover, inclusion of an interaction term between Weekly OA and gender into the model (i.e., a test of moderation of Weekly OA by gender) was not statistically significant (ΔR^2^ = 0.007, Wald *Χ*^2^_(3)_ = 1.327, p = 0.723).

**Table 5:** Categorical polytomous BMI outcomes regressed upon Occupational Activity^§^ among employed individuals participating in the HCHS/SOL study, stratified by gender (N = 7,354^a^).

		Overall			Female*				Male*			
		(N = 7,354)			(N = 3,948)				(N = 3,406)			
	Prev^b^	AOR	95% CI	p-value	Prev^b^	AOR	95% CI	p-value	Prev^b^	AOR	95% CI	p-value
**Underweight** (< 18.5)	0.76	1.051	(0.971, 1.138)	0.218	0.90	1.056	(0.962, 1.159)	0.253	0.65	1.083	(0.954, 1.229)	0.218
**Normal** (18.5–24.9)	22.13	1	1	…	24.25	1	1	…	20.47	1	1	…
**Overweight** (25.0–29.9)	39.23	1.038	(1.021, 1.056)	< 0.0001	34.18	1.011	(0.982, 1.040)	0.463	43.17	1.045	(1.018, 1.072)	0.001
**Obese** (≥ 30)	37.89	1.032	(1.014, 1.050)	< 0.001	40.66	1.011	(0.986, 1.037)	0.377	35.72	1.040	(1.013, 1.068)	0.004

^§^ Occupational Activity = 10 MET x hours x week^-1^

* Test for moderation of OA by gender (i.e. OA*Gender) was not statistically significant (ΔR^2^ = 0.007, Wald *Χ*^2^_(3)_ = 1.327, p = 0.723).

AOR—Adjusted Odds Ratio.

Variables forced into the model as covariates: Age, Income, Background, Acculturation, Cigarette Use, Energy Consumed, Transportation Physical Activity, and Leisure Time Physical Activity.

MET equivalent for occupation “Other” was the population based weighted mean of all other occupations.

^a.^ 7,354 individuals had non-missing values for OA and all covariates.

^b.^ Adjusted prevalence.

Further exploration of the results presented in Table 5 led to the examination of the relative contribution of each of the components of Weekly OA, namely Total Hours Worked and occupational METs, to the outcome. When polytomous BMI categories were regressed upon Weekly OA, controlling for Total Hours Worked, Weekly OA was no longer a statistically significant predictor of BMI outcomes neither for the overall population nor for males. However, when an identical model was analyzed, controlling for occupational METs instead of Total Hours Worked, we observed outcomes analogous to those presented in Table 5, i.e., Weekly OA maintaining statistical significance for the overall population and for males. The results of this component exploration suggested an additional need to examine the association of BMI and Total Hours Worked.

Table 6 demonstrates the results of the polytomous BMI categorical outcomes regressed upon Total Hours Worked. After adjusting for the covariates, the estimated overall adjusted odds for being overweight were 16.3% greater than the odds of being normal weight for each additional 10 hrs/wk of work (AOR = 1.16, 95% CI 1.01, 1.24). Similarly, there was a 14.4% increased odds of being obese compared to normal weight for each additional 10 hrs/wk of Total Hours Worked (AOR = 1.14, 95% CI 1.07, 1.23). Males exhibited trends similar to the overall population in that the odds of being overweight or obese, vs. normal weight, for each increasing 10 hrs/wk of Total Hours Worked was 24.5% (AOR = 1.25, 95% CI 1.14, 1.36) and 19.0% (AOR = 1.19, 95% CI 1.08, 1.32) greater, respectively. There were no significant associations for females when examining the relationship between BMI and Total Hours Worked.

**Table 6:** Categorical polytomous BMI outcomes regressed upon Total Hours Worked^§^ among employed individuals participating in the HCHS/SOL study, stratified by gender (N = 7,354^a^).

		Overall			Female*				Male*			
		(N = 7,354)			(N = 3,948)				(N = 3,406)			
	Prev^b^	AOR	95% CI	p-value	Prev^b^	AOR	95% CI	p-value	Prev^b^	AOR	95% CI	p-value
**Underweight** (< 18.5)	0.57	1.058	(0.902, 1.488)	0.2150	0.61	1.238	(0.983, 1.561)	0.070	0.53	1.145	(0.729, 1.798)	0.556
**Normal** (18.5–24.9)	19.87	1	1	…	20.95	1	1	…	18.61	1	1	…
**Overweight** (25.0–29.9)	40.01	1.163	(1.093, 1.237)	< 0.0001	35.89	0.997	(0.905, 1.099)	0.952	44.77	1.245	(1.138, 1.362)	< 0.0001
**Obese** (≥ 30)	39.56	1.144	(1.068, 1.226)	< 0.001	42.55	1.056	(0.955, 1.168)	0.288	36.08	1.191	(1.078, 1.315)	< 0.001

^§^ Total Hours Worked = 10 hours x week^-1^. Where: Total hours worked = (Primary Occupation (hrs/wk) + (Secondary Occupation (hrs/wk)).

AOR—Adjusted Odds Ratio.

Variables forced into the model as covariates: Age, Income, Background, Acculturation, Cigarette Use, Energy Consumed, Transportation Physical Activity, and Leisure Time Physical Activity.

^a.^ 7,354 individuals had non-missing values for total hours worked and all covariates.

^b.^ Adjusted prevalence.

## Discussion

The HCHS/SOL represents a rich data set derived from a population-based prospective cohort, multi-site study that permits the evaluation of multiple health outcomes among Hispanic/Latino individuals. Our study represents one of the first attempts to characterize the association between overweight/obesity and occupational parameters in a Hispanic/Latino population. By classifying our sample into BMI categories, we were able to examine population-estimated associations with Employment Status, SROA, Weekly OA, and Total Hours Worked. We found that increasing Weekly OA and Total Hours Worked for the overall population and for males were significantly associated with being overweight and obese. Further analysis indicated that Total Hours Worked might be the most important indicator of risk of being overweight/obese.

The prevalence of overweight/obesity in our cross-sectional analysis of employed U.S. Hispanic/Latino individuals in this study (77.1%) was greater than that reported by Ogden et al. for the U.S. adult population (69%), yet is consistent with their findings for Hispanic individuals (77.1%) [39]. Our study found no association between Employment Status and overweight/obese status. This suggests that after taking into account the socioeconomic benefits attributed to being employed, employment was neither a protective nor a risk factor for overweight/obesity. In contrast, Martin et al., [22] found a higher risk of obesity among unemployed vs. actively employed individuals. It may be noteworthy that among those employed, Martin et al. [22] distinguished between those who worked from home and those who did not, a distinction not present in the current study. Although there was a low prevalence of underweight in our study, our findings suggested that employed individuals were less likely to be underweight compared to normal weight. While there is sparse literature regarding the association between employment status and underweight, our finding is in agreement with Ali and Lindstrom [40] who found that among Swedish women aged 18–34 years, employment was less common among underweight, compared to normal weight women. Moreover, Imai et al. [41] found that disability was more common among underweight persons of both genders compared to normal weight individuals. By extension, being underweight may be negatively associated with employment due to the effects of poor health and disability rather than BMI.

Our lack of significant findings with the SROA data, which are in line with findings from Chau et al. [42], contrast with our findings that used constructed measures of Weekly OA. When we examined the association of Weekly OA and overweight/obesity, for our overall population, as well as males, we observed a positive association with the adjusted odds of being either overweight or obese compared to normal weight. The more occupationally active participants were, the greater their adjusted likelihood of being overweight or obese. Such results appear to be counterintuitive from the perspective of energy balance considerations. However, there were several factors that we were only partially account for (i.e., LTPA and TPA) and that may have contributed to an overall positive energy balance. In contrast to our study, previous studies [27,42,43] have demonstrated a higher association of overweight and obesity with low activity occupations compared to high activity occupations.

The unadjusted associations of LTPA with BMI categories in our study demonstrated a greater proportion of overweight and obese individuals who reported no LTPA, and a smaller proportion of overweight and obese individuals who reported vigorous LTPA compared to normal weight individuals (S2 Table), consistent with 2004–2006 NHANES data as reported by Archer et al. [44]. Such observations may underscore the potential importance of LTPA as a determinant of overweight/obesity and highlight the need to explore the dynamics of the relationships among Weekly OA, Total Hours Worked, and LTPA.

In light of these considerations, we explored the impact of the Total Hours Worked per week on overweight/obesity. Our results demonstrated that for both overall population and for males, adjusted odds of being overweight or obese compared to normal weight, increased significantly for each 10-hour increment of work per week, whether in one or more than one occupation. Similarly, Luckhaupt et al. [45] and Jang et al. [46] reported significant associations of long work hours with obesity for all workers, and for males employed in “manual” occupations, respectively. These findings support the observation, that working extended hours, independent of the physical activity attributable to the occupation, is the critical factor linking occupation and overweight/obesity. Perhaps, it is that long work hours “crowd out” time and motivation available for LTPA.

Access to LTPA, as a consistent part of one’s lifestyle, may be dependent upon available time and money, both of which are likely to accrue through higher education and income. Bonauto et al. [43] found that workers with higher SES tended to have healthier behaviors than those with lower SES. Kaleta et al. [47] reported significantly increased risk of obesity both among workers with a primary education compared to university-educated workers, and among low income workers compared to high-income workers, yet no association of BMI with OA. McLaren [48] demonstrated that the socioeconomic disparities associated with increased risk of obesity may be explained by higher educational attainment and income, which may correlate with higher expectations specific to personal health, appearance, and adequate economic capacity to purchase healthy but more expensive food items, and increased opportunities for LTPA.

We found that employed participants in the HCHS/SOL tended to have low income levels and almost 55% reported no LTPA. Trivedi, et al. [49] reported that a higher prevalence of obesity among rural residents was accompanied by higher prevalence of no LTPA and failure to meet recommended LTPA levels compared to urban dwellers. Moreover, Caban-Martinez et al. [50] found a lower prevalence of recommended LTPA levels reported among Hispanic workers compared to non-Hispanic workers. Such observations seem to suggest that LTPA may be a critical factor in clarifying the relationship between total hours worked and BMI. The demands of occupations requiring long work hours, may limit participation in LTPA in a multifactorial manner, owing to diminished physical capacity after a demanding work schedule, lack of free time to engage in such activities, or limited economic resources that preclude participation in LTPA.

## Strengths and Limitations

One of the strengths of this study is that the HCHS/SOL is a large, population-based cohort that used a sampling method that permits findings that are free of outcome-based selection bias. Probability sampling of the participants, from communities selected for Hispanic/Latino background diversity, allow for inferences beyond the selected sample to urban dwelling Hispanic/Latino populations within the four geographical regions [30]. However, the cross-sectional nature of the study design and data analysis precludes provision of temporal relations or causal inferences for the associations found. Additionally, selected variables in the analyses are based upon self-report and, as such, differential recall may have been a factor affecting data by under- or over-reporting.

An additional strength of our findings is that the measures used to estimate Weekly OA were derived from detailed, population-based MET estimates assigned to occupations [34], as opposed to self-report data. The estimates of energy consumption were collected via methods that have been validated in comparison to objective measures by some authors [37,38] yet questioned by others [11,12,51]. Although our multivariable analyses attempted to apply energy balance considerations, quantative measures of several PA domains were missing, restricting the control of a comprehensive energy balance equation. An important direction for future research could be to combine objective PA assessment (e.g., accelerometer) with a diary of PA domains over the same time period. This way the objective measure of PA levels can be better separated by activity domain (OA, LTPA, TPA, lifestyle, etc.) and provide a more comprehensive examination of energy expenditure.

## Conclusions

This study presents the first findings on the association between OA with overweight/obesity among Hispanics/Latinos in the U.S. The size and rapid growth of this segment of the population, combined with the high prevalence of overweight (39%) and obesity (38%) reported for employed individuals in this study, underscores the importance of better identifying and understanding the determinants of the obesity epidemic among U.S. Hispanics/Latinos. The finding that increasing Weekly OA was associated with increasing odds of overweight and obesity compared to normal weight suggests that the workplace is only one part of the overall energy expenditure dynamic contributing to overweight/obesity. Moreover, our finding that the Total Hours Worked may be the critical factor in workplace, rather than the activity level of the particular occupation, underscores that time in the workplace may impact energy expenditure outside of work, particularly with respect to LTPA. An implication of this study, consistent with the goals of Healthy People 2020 to increase health promotion programs in the workplace [52] is to increase workplace interventions and target occupations where risk of obesity is greatest. Perhaps, targeting individuals in “high-risk” occupations that require especially long work hours, by promoting and incentivizing LTPA interventions, may prove to be effective instruments to combat the obesity epidemic.

## Supporting Information

S1 Table Occupational Categories. (DOCX)

S2 Table Unadjusted bivariate associations within BMI categories and characteristics of employed individuals participating in the Hispanic Community Health Study/Study of Latinos (HCHS/SOL), United States 2008–2011 (n = 7,409). Unadjusted column percent (95% CL), Mean (SE).
(DOCX)
