# Supplementary material for: Occupational Physical Activity and Body Mass Index: Results from the Hispanic Community Health Study / Study of Latinos
Source: PLoS One. 2016 Mar 31;11(3):e0152339. doi: 10.1371/journal.pone.0152339 (PMC4816339; doi:10.1371/journal.pone.0152339)
Supplement: S1 Table — (DOCX) [file pone.0152339.s001.docx]

**S1 Table. Occupational Categories**

| **Occupational Category** | **Description** |
| --- | --- |
| 1 | Senior professional/technical worker (doctor, professor, lawyer, architect, engineer) |
| 2 | Junior professional/Technical worker (midwife, nurse, teacher, editor, photographer) |
| 3 | Administrator/executive/manager (working proprietor, government official, section chief, department or bureau director, administrative cadre, village leader) |
| 4 | Office staff (secretary, office helper) |
| 5 | Farmer, fisherman, hunter |
| 6 | Skilled worker (foreman, group leader, craftsman) |
| 7 | Non-skilled worker (ordinary laborer, construction, yard, migrant laborer) |
| 8 | Army officer, police officer |
| 9 | Ordinary soldier, policeman |
| 10 | Driver (transportation of goods from one location to another) |
| 11 | Service worker (housekeeper, cook, waiter, doorkeeper, hairdresser, counter salesperson, launderer, child care worker) |
| 12 | Athlete, actor, musician |
| 13 | Other |
| 99 | Don’t know/refused |
