# Supplementary material for: Occupational Physical Activity and Body Mass Index: Results from the Hispanic Community Health Study / Study of Latinos
Source: PLoS One. 2016 Mar 31;11(3):e0152339. doi: 10.1371/journal.pone.0152339 (PMC4816339; doi:10.1371/journal.pone.0152339)
Supplement: S2 Table — Unadjusted column percent (95% CL), Mean (SE). (DOCX) [file pone.0152339.s002.docx]

| **S2 Table.** **Unadjusted bivariate associations within BMI categories and characteristics of employed individuals participating in the Hispanic Community Health Study/Study of Latinos (HCHS/SOL), United States 2008–2011 (n=7,409). Unadjusted column percent (95% CL), Mean (SE).** | | | | | | | | | |
| --- | --- | --- | --- | --- | --- | --- | --- | --- | --- |
|  | **Underweight** | |  | **Normal** |  | **Overweight** |  | **Obese** |  |
|  | BMI < 18.5 | |  | BMI = 18.5–24.9 |  | BMI = 25.0–29.9 |  | BMI ≥ 30 |  |
| **CHARACTERISTIC** | (N = 42) | |  | (N = 1475) |  | (N = 2967) |  | (N = 2925) |  |
|  | Col. % (95% CL) | | p-value | Col. % (95% CL) | p-value | Col. % (95% CL) | p-value | Col. % (95% CL) | p-value |
|  | Mean (SE) | |  | Mean (SE) |  | Mean (SE) |  | Mean (SE) |  |
| **GENDER** |  | |  |  |  |  |  |  |  |
| Male | 47.84 (32.70, 62.98) | | 0.588 | 52.17 (48.61, 55.73) | Reference | 61.91 (59.40, 64.42) | < 0.0001 | 52.90 (50.08, 55.72) | 0.757 |
| Female | 52.16 (37.02, 67.30) | |  | 47.83 (44.27, 51.39) |  | 38.09 (35.58, 40.60) |  | 47.10 (44.28, 49.92) |  |
|  |  | |  |  |  |  |  |  |  |
| **AGE** | 29.48 (2.04) | | 0.006 | 35.32 (0.45) | Reference | 40.62 (0.38) | < 0.0001 | 39.52 (0.34) | < 0.0001 |
| 18 – 29 years | 65.60 (50.88, 80.32) | | 0.058^a^ | 41.53 (37.79, 45.26) | Reference | 20.85 (18.28, 23.42) | < 0.0001 | 23.97 (21.25, 26.69) | < 0.0001 |
| 30 – 39 years | 11.70 (0.71, 22.69) | |  | 22.87 (19.76, 25.98) |  | 26.80 (23.99, 29.62) |  | 26.71 (24.02, 29.39) |  |
| 40 – 49 years | 12.76 (2.16, 23.36) | |  | 18.98 (16.29, 21.68) |  | 27.57 (25.19, 29.94) |  | 26.89 (24.75, 29.04) |  |
| 50 – 59 years | 8.30 (0.00, 18.90) | |  | 10.63 (9.07, 12.19) |  | 17.45 (15.73, 19.17) |  | 16.92 (15.22, 18.62) |  |
| 60 – 69 years | 1.65 (0.00, 4.04) | |  | 5.41 (3.99, 6.82) |  | 6.45 (5.29, 7.62) |  | 5.13 (4.11, 6.14) |  |
| 70 - 74 years | --- | |  | 0.58 (0.09, 1.07) |  | 0.88 (0.40, 1.36) |  | 0.38 (0.10, 0.66) |  |
|  |  | |  |  |  |  |  |  |  |
| **EDUCATION** |  | |  |  |  |  |  |  |  |
| < HS | 4.74 (0.00, 12.44) | | 0.406 | 11.21 (9.25, 13.17) | Reference | 16.57 (14.64, 18.51) | 0.003 | 15.69 (13.71, 17.67) | 0.033 |
| HS (or Preparatory School) | 49.44 (35.24, 63.65) | |  | 40.47 (36.95, 43.99) |  | 38.85 (35.85, 41.86) |  | 38.41 (35.37, 41.45) |  |
| Trade or Vocational School | 5.68 (0.00, 12.22) | |  | 10.41 (8.44, 12.39) |  | 11.80 (10.01, 13.59) |  | 11.57 (9.71, 13.43) |  |
| University or College | 39.33 (24.07, 54.58) | |  | 35.96 (32.05, 39.86) |  | 31.34 (28.37, 34.31) |  | 32.66 (29.75, 35.57) |  |
| Other | 0.82 (0.00, 2.46) | |  | 1.95 (1.06, 2.84) |  | 1.43 (0.72, 2.15) |  | 1.66 (0.99, 2.33) |  |
|  |  | |  |  |  |  |  |  |  |
| **HOUSEHOLD INCOME** |  | |  |  |  |  |  |  |  |
| < $10,000 | 12.51 (3.13, 21.89) | | 0.551 | 7.23 (5.47, 8.99) | Reference | 6.97 (5.56, 8.39) | 0.443 | 7.62 (6.29, 8.96) | 0.895 |
| $10,001 - 20,000 | 31.57 (11.40, 51.74) | |  | 25.50 (22.11, 28.90) |  | 29.56 (26.61, 32.51) |  | 27.16 (24.59, 29.74) |  |
| $20,001 - 40,000 | 27.37 (13.67, 41.07) | |  | 39.38 (35.61, 43.15) |  | 37.47 (34.76, 40.18) |  | 37.92 (35.23, 40.61) |  |
| $40,001 - 75,000 | 23.75 (2.06, 45.45) | |  | 19.68 (16.35, 23.02) |  | 18.33 (16.13, 20.54) |  | 18.80 (16.44, 21.17) |  |
| > $75,000 | 4.79 (0.00, 11.81) | |  | 8.20 (6.06, 10.34) |  | 7.66 (5.18, 10.15) |  | 8.49 (6.77, 10.21) |  |
|  |  | |  |  |  |  |  |  |  |
| **MARITAL STATUS** |  | |  |  |  |  |  |  |  |
| Single | 61.03 (44.83, 77.22) | | 0.038 | 43.70 (40.13, 47.27) | Reference | 28.13 (25.21, 31.06) | < 0.0001 | 31.39 (28.54, 34.24) | < 0.0001 |
| Married or living with a partner | 36.69 (20.92, 52.46) | |  | 42.23 (38.69, 45.78) |  | 57.47 (54.36, 60.57) |  | 55.87 (52.85, 58.89) |  |
| Separated, divorced, or widow(er) | 2.28 (0.00, 6.80) | |  | 14.07 (11.74, 16.39) |  | 14.40 (12.68, 16.12) |  | 12.74 (11.02, 14.45) |  |
|  |  | |  |  |  |  |  |  |  |
| **BACKGROUND** |  | |  |  |  |  |  |  |  |
| Dominican | 12.25 (0.02, 24.48) | | 0.987 | 8.72 (6.45, 10.99) | Reference | 9.42 (7.64, 11.19) | 0.3095 | 9.79 (7.57, 12.01) | 0.0581 |
| Central American | 10.61 (1.36, 19.86) | |  | 8.66 (6.40, 10.93) |  | 8.48 (6.89, 10.06) |  | 7.74 (6.15, 9.34) |  |
| Cuban | 16.47 (1.31, 31.63) | |  | 15.52 (11.84, 19.20) |  | 15.40 (12.13, 18.67) |  | 15.77 (12.30, 19.24) |  |
| Mexican | 39.12 (19.37, 58.86) | |  | 41.67 (36.98, 46.37) |  | 45.56 (40.90, 50.23) |  | 42.58 (38.78, 46.37) |  |
| Puerto Rican | 10.93 (0.51, 21.35) | |  | 13.12 (10.57, 15.66) |  | 10.12 (8.05, 12.20) |  | 15.17 (12.91, 17.44) |  |
| South American | 5.65 (0.00, 12.14) | |  | 7.74 (5.79, 9.69) |  | 6.51 (5.23, 7.78) |  | 4.55 (3.51, 5.58) |  |
| More than one/Other heritage | 4.97 (0.00, 13.06) | |  | 4.57 (3.02, 6.12) |  | 4.51 (3.15, 5.87) |  | 4.40 (2.90, 5.90) |  |
|  |  | |  |  |  |  |  |  |  |
| **ACCULTURATION** |  | |  |  |  |  |  |  |  |
| Language Subscale | 2.40 (0.17) | | 0.376 | 2.24 (0.04) | Reference | 2.02 (0.03) | < 0.0001 | 2.23 (0.04) | 0.812 |
| Language Subscale | 2.34 (0.09) | | 0.730 | 2.30 (0.02) | Reference | 2.22 (0.02) | < 0.001 | 2.29 (0.02) | 0.650 |
|  |  | |  |  |  |  |  |  |  |
|  |  | |  |  |  |  |  |  |  |
|  |  | |  |  |  |  |  |  |  |
|  |  | |  |  |  |  |  |  |  |
| **FIELD CENTER** |  | |  |  |  |  |  |  |  |
| Bronx | 19.63 (4.58, 34.67) | | 0.970 | 23.13 (19.16, 27.11) | Reference | 24.52 (20.99, 28.06) | 0.624 | 28.40 (24.76, 32.04) | 0.031 |
| Chicago | 18.68 (3.07, 34.28) | |  | 19.64 (16.24, 23.04) |  | 19.66 (16.78, 22.54) |  | 20.87 (18.20, 23.55) |  |
| Miami | 28.27 (9.36, 47.18) | |  | 27.80 (22.98, 32.63) |  | 25.44 (21.14, 29.73) |  | 24.22 (19.87, 28.57) |  |
| San Diego | 33.43 (5.70, 61.16) | |  | 29.42 (24.46, 34.39) |  | 30.38 (25.17, 35.59) |  | 26.50 (22.76, 30.25) |  |
|  |  | |  |  |  |  |  |  |  |
| **EMPLOYMENT STATUS^b^** |  | |  |  |  |  |  |  |  |
| Not currently employed | 67.35 (55.99, 78.72) | | 0.003 | 48.78 (46.34, 51.21) | Reference | 47.72 (45.55, 49.88) | 0.502 | 53.09 (51.25, 54.94) | 0.004 |
| Employed full / part-time | 32.65 (21.28, 44.01) | |  | 51.22 (48.79, 53.66) |  | 52.28 (50.12, 54.45) |  | 46.91 (45.06, 48.75) |  |
|  |  | |  |  |  |  |  |  |  |
| **PRIMARY OCCUPATION** |  | |  |  |  |  |  |  |  |
| Office staff | --- | | c | 5.52 (3.74, 7.30) | Reference | 4.25 (3.18, 5.32) | 0.047 | 5.43 (3.80, 7.05) | 0.007 |
| Senior professional/technical worker | 4.42 (0.00, 13.02) | |  | 2.64 (1.44, 3.83) |  | 1.77 (1.19, 2.35) |  | 1.51 (0.88, 2.14) |  |
| Administrator/executive/manager | 3.91 (0.00, 10.25) | |  | 2.87 (1.90, 3.84) |  | 4.08 (2.15, 6.01) |  | 4.60 (3.50, 5.70) |  |
| Driver | --- | |  | 1.82 (0.93, 2.71) |  | 3.18 (2.18, 4.18) |  | 2.72 (1.91, 3.53) |  |
| Athlete, actor, musician | 1.70 (0.00, 5.10) | |  | 0.83 (0.00, 1.69) |  | 0.90 (0.05, 1.74) |  | 0.07 (0.00, 0.17) |  |
| Junior professional/Technical worker | 1.44 (0.00, 4.26) | |  | 2.67 (1.57, 3.78) |  | 3.17 (2.31, 4.02) |  | 2.86 (1.93, 3.79) |  |
| Army officer, police officer | --- | |  | 0.23 (0.00, 0.60) |  | 0.15 (0.00, 0.34) |  | 0.34 (0.05, 0.63) |  |
| Ordinary soldier, policeman | --- | |  | d |  | 0.32 (0.00, 0.70) |  | 0.14 (0.00, 0.31) |  |
| Service worker | 22.97 (10.56, 35.39) | |  | 20.26 (17.18, 23.35) |  | 15.14 (13.39, 16.89) |  | 18.32 (16.04, 20.59) |  |
| Skilled worker | 14.92 (3.25, 26.60) | |  | 21.25 (18.01, 24.49) |  | 24.40 (21.98, 26.81) |  | 23.47 (21.24, 25.69) |  |
| Other^e^ | 19.95 (4.62, 35.28) | |  | 12.99 (10.33, 15.64) |  | 12.27 (10.21, 14.33) |  | 12.99 (10.90, 15.08) |  |
| Non-skilled worker | 30.67 (14.66, 46.68) | |  | 27.38 (24.05, 30.71) |  | 29.51 (26.74, 32.28) |  | 26.83 (24.35, 29.31) |  |
| Farmer, fisherman, hunter | --- | |  | 0.02 (0.00, 0.07) |  | 0.02 (0.00, 0.07) |  | 0.11 (0.00, 0.22) |  |
| Don’t know/refused | --- | |  | 1.51 (0.68, 2.35) |  | 0.85 (0.32, 1.39) |  | 0.62 (0.30, 0.95) |  |
|  |  | |  |  |  |  |  |  |  |
| **OCCUPATIONAL MET CATEGORIES** |  | |  |  |  |  |  |  |  |
| Category 1 1.00 - 1.99 METS | 8.33 (0.00, 19.00) | | 0.648 | 13.05 (10.35, 15.74) | Reference | 13.40 (11.02, 15.77) | 0.773 | 14.34 (12.18, 16.50) | 0.880 |
| Category 2 2.00 - 2.99 METS | 41.05 (26.24, 55.86) | |  | 45.94 (42.54, 49.35) |  | 44.44 (41.75, 47.14) |  | 45.48 (42.79, 48.18) |  |
| Category 3 3.00 - 3.99 METS | 19.95 (4.62, 35.28) | |  | 13.19 (10.50, 15.88) |  | 12.37 (10.30, 14.45) |  | 13.07 (10.97, 15.17) |  |
| Category 4 >= 4.00 METS | 30.67 (14.66, 46.68) | |  | 27.82 (24.45, 31.20) |  | 29.79 (26.97, 32.60) |  | 27.11 (24.60, 29.61) |  |
|  |  | |  |  |  |  |  |  |  |
| **PRIMARY OCCUPATION** (hrs/wk) | 35.54 (2.70) | | 0.924 | 35.28 (0.48) | Reference | 37.37 (0.34) | < 0.001 | 37.05 (0.39) | 0.004 |
|  |  | |  |  |  |  |  |  |  |
| **SECONDARY OCCUPATION** (hrs/wk) | 15.20 (2.43) | | 0.362 | 12.69 (1.03) | Reference | 15.55 (1.03) | 0.062 | 14.41 (1.01) | 0.218 |
|  |  | |  |  |  |  |  |  |  |
| **TOTAL HOURS WORKED** (hrs/wk) | 38.39 (2.43) | | 0.412 | 36.31 (0.50) | Reference | 39.13 (0.37) | < 0.0001 | 38.54 (0.40) | < 0.001 |
|  |  | |  |  |  |  |  |  |  |
| **EMPLOYMENT STATUS** |  | |  |  |  |  |  |  |  |
| Part-time (< 40 hrs/wk) | 37.30 (23.23, 51.37) | | 0.328 | 44.84 (41.06, 48.62) | Reference | 36.38 (33.93, 38.84) | < 0.001 | 39.08 (36.27, 41.86) | 0.014 |
| Full-Time (≥ 40 hrs/wk) | 62.70 (48.63, 76.77) | |  | 55.16 (51.38, 58.94) |  | 63.62 (61.16, 66.07) |  | 60.92 (58.14, 63.71) |  |
|  |  | |  |  |  |  |  |  |  |
| **PHYSICAL ACTIVITY** |  | |  |  |  |  |  |  |  |
| Leisure Time (≥ 10 continuous min) |  | |  |  |  |  |  |  |  |
| None | 54.81 (40.57, 69.05) | | 0.210 | 49.18 (45.52, 52.84) | Reference | 53.44 (50.24, 56.64) | 0.027 | 59.00 (56.01, 61.98) | < 0.0001 |
| Moderate | 23.46 (10.02, 36.91) | |  | 14.78 (12.59, 16.98) |  | 15.90 (14.25, 17.55) |  | 17.08 (15.01, 19.15) |  |
| Vigorous | 11.59 (0.36, 22.81) | |  | 18.74 (15.30, 22.18) |  | 13.70 (11.67, 15.73) |  | 9.93 (7.95, 11.90) |  |
| Moderate & Vigorous | 10.14 (1.84, 18.43) | |  | 17.30 (14.35, 20.24) |  | 16.96 (14.64, 19.28) |  | 13.99 (11.74, 16.25) |  |
| Occupational Energy Expenditure (kcal/wk) | 6,383.75 (606.03) | | 0.215 | 7,162.05 (125.73) | Reference | 9,636.28 (128.05) | < 0.0001 | 11,708.00 (158.60) | < 0.0001 |
| Occupational Activity (MET*hrs/wk) | 122.89 (11.95) | | 0.308 | 110.48 (1.81) | Reference | 121.08 (1.58) | < 0.0001 | 117.34 (1.48) | 0.002 |
| Self-Reported Occupational Activity (hrs/wk) | 28.48 (6.45) | | 0.186 | 37.15 (1.89) | Reference | 37.85 (1.30) | 0.755 | 39.59 (1.45) | 0.298 |
| Transportation (Walk or bicycle ≥ 10 min) |  | |  |  |  |  |  |  |  |
| No | 57.96 (41.22, 74.69) | | 0.607 | 53.49 (49.50, 57.49) | Reference | 54.96 (51.80, 58.13) | 0.541 | 56.07 (53.23, 58.91) | 0.268 |
| Yes | 42.04 (25.31, 58.78) | |  | 46.51 (42.51, 50.50) |  | 45.04 (41.87, 48.20) |  | 43.93 (41.09, 46.77) |  |
|  |  | |  |  |  |  |  |  |  |
| **DIETARY COMPOSITION** |  | |  |  |  |  |  |  |  |
| Total Energy Consumption (kcal/day) | 2,212.48 (88.76) | | 0.246 | 2,107.41 (18.18) | Reference | 2,101.37 (14.28) | 0.780 | 2,025.11 (15.11) | < 0.001 |
| **CIGARETTE USE** |  | |  |  |  |  |  |  |  |
| Never | 60.35 (40.51, 80.19) | | 0.122 | 68.72 (65.22, 72.22) | Reference | 65.08 (62.47, 67.70) | 0.001 | 60.50 (57.74, 63.26) | < 0.0001 |
| Former | 6.89 (0.00, 14.88) | |  | 12.37 (10.12, 14.62) |  | 18.95 (16.99, 20.92) |  | 19.86 (17.84, 21.88) |  |
| Current | 32.76 (16.54, 48.97) | |  | 18.91 (15.84, 21.99) |  | 15.96 (13.87, 18.05) |  | 19.64 (17.16, 22.11) |  |
|  | |  |  |  |  |  |  |  |  |

Notes: The analytic sample includes those participants of the HCHS/SOL who are employed with non-missing data for Age, BMI, Income, Background, Acculturation, Cigarette Use, Energy Consumed, Transportation Physical Activity, and Leisure Time Physical Activity (N = 7,409, prevalence = 45.14%).

a. Ages 70 - 74 years excluded.

b. Employment Status associations based upon the complete HCHS/SOL sample (N = 16,415).

c. Multiple cells with missing values or zero values, chi square not calculated.

d. Occupational categorie 8 missing, combined with category 9.

e. MET equivalent for occupation “Other” was the population based weighted mean of all other occupations.
